# Disparities and relative risk ratio of preterm birth in six Central and Eastern European centers

**DOI:** 10.3325/cmj.2015.56.119

**Published:** 2015-04

**Authors:** Chander P Arora, Marian Kacerovsky, Balazs Zinner, Tibor Ertl, Iuliana Ceausu, Igor Rusnak, Serhiy Shurpyak, Meenu Sandhu, Calvin J Hobel, Daniel A Dumesic, Sandor G Vari

**Affiliations:** 1 International Research and Innovation Management Program, Cedars-Sinai Medical Center, Los Angeles, CA, USA; 2 Department of Obstetrics and Gynecology, Charles University, Prague and Faculty of Medicine and University Hospital, Hradec Kralove, Czech Republic; 3 Department of Obstetrics and Gynecology, Semmelweis University, Budapest, Hungary; 4 Department of Obstetrics and Gynecology, Medical School, University of Pecs, Pecs, Hungary; 5 Department of Obstetrics and Gynecology, Carol Davila University of Medicine and Pharmacy, Bucharest, Romania; 6 Department of Obstetrics and Gynecology, Slovak Medical University Hospital, Bratislava, Slovak Republic; 7 Department of Obstetrics and Gynecology and Perinatology, Danylo Halytskyy Lviv National Medical University, Lviv, Ukraine; 8 Department of Obstetrics and Gynecology, David Geffen School of Medicine, University of California, Los Angeles, CA, USA; * BZ, TE, IC, IR have equal contributions.

## Abstract

**Aim:**

To identify characteristic risk factors of preterm birth in Central and Eastern Europe and explore the differences from other developed countries.

**Method:**

Data on 33 794 term and 3867 preterm births (<37 wks.) were extracted in a retrospective study between January 1, 2007 and December 31, 2009. The study took place in 6 centers in 5 countries: Czech Republic, Hungary (two centers), Romania, Slovakia, and Ukraine. Data on historical risk factors, pregnancy complications, and special testing were gathered. Preterm birth frequencies and relevant risk factors were analyzed using Statistical Analysis System (SAS) software.

**Results:**

All the factors selected for study (history of smoking, diabetes, chronic hypertension, current diabetes, preeclampsia, progesterone use, current smoking, body mass index, iron use and anemia during pregnancy), except the history of diabetes were predictive of preterm birth across all participating European centers. Preterm birth was at least 2.4 times more likely with smoking (history or current), three times more likely with preeclampsia, 2.9 times more likely with hypertension after adjusting for other covariates. It had inverse relationship with the significant predictor body mass index, with adjusted risk ratio of 0.8 to 1.0 in three sites. Iron use and anemia, though significant predictors of preterm birth, indicated mixed patterns for relative risk ratio.

**Conclusion:**

Smoking, preeclampsia, hypertension and body mass index seem to be the foremost risk factors of preterm birth. Implications of these factors could be beneficial for design and implementation of interventions and improve the birth outcome.

Preterm birth (PTB: spontaneous and indicated), defined as delivery before 37 weeks of gestation, is the most common cause of neonatal mortality in developed countries (1). Worldwide, prematurity complications are the most common cause of neonatal deaths (2), accounting for 80% of the world’s 1.1 million deaths (3,4). Fetal, neonatal, and infant mortality rates vary widely between the countries of Europe. Preterm babies born before 28 weeks of gestational age constitute over one-third of all deaths, but data are not comparable between countries (5).

Children born prematurely have a higher incidence of cerebral palsy, sensory deficits, respiratory illnesses, and learning disabilities compared to children born at term. The morbidity associated with preterm birth often extends to later life, resulting in enormous physical, psychological, and economic costs (6,7).

In developing countries, accurate and complete population data and medical records often do not exist. Therefore, few international studies regarding preterm birth and neonatal deaths have compared social, economic, or ethnic differences, even though several potential risk factors for preterm birth have been identified, including race, physical environment, nutrition, socioeconomic status, and reproductive age (1,8). Maternal height and weight may also contribute to PTB (9-12), along with cigarette smoking or illicit drug use (13,14). In addition, maternal iron, folic acid, and vitamin D deficiencies may increase the risk for preterm delivery, with nutritional differences among ethnic groups likely contributing to disparities in prematurity (15).

The contribution of specific risk factors for PTB in Central and Eastern European counties is unknown. Therefore, the Mother and Child Health Research Network (M&CH RN), as one of the networks of the Association for Regional Cooperation in Health, Science and Technology (RECOOP HST), instituted a retrospective review of delivery records of participating hospitals to identify the risk factors of preterm birth. Its specific aims were to identify the risk factors of preterm birth specific for Central and Eastern Europe (CEE).

## Population and methods

### Study strategy

Eight sites from seven member countries of the M&CH RN were included in the exploratory retrospective study. During the study, two of the centers in Croatia and Poland were removed from the study as they failed to meet the study criteria, mainly due to incomplete data review after the data extraction. Clinical data from 37 661 records of singleton deliveries (vaginal or cesarean section) were extracted for preterm births (<37 wks.) and full-term births (>37 wks.) between 2007 and 2009 in 6 centers of 5 countries: Czech Republic (University Hospital, Hradec Kralove), Hungary (Budapest, Semmelweis University; and Pecs, Medical School, University of Pecs, a regional tertiary center for preterm birth), Romania (Carol Davila University of Medicine and Pharmacy), Slovakia (Slovak Medical University Hospital), and Ukraine (Danylo Halytskyy Lviv National Medical University). The center at Pecs contributed data for two years, 2007-2008. Each center acquired the Ethics Committee approval from their respective institutions.

Data collection sheets covering 94 parameters distributed among three modules were created collaboratively and approved by each center. The three modules relevant to preterm birth were designed to collect information on: (i) historical risk factors, such as socioeconomic factors, smoking, drug use, and diseases (46 parameters); (ii) problems and interventions in the current pregnancy like vaginal bleeding and cerclage (27 parameters); and (iii) special tests and measurement, such as chorionic villi sampling or progesterone use (21 parameters). The data were entered into an online electronic data entry form using a web based data system (Flexi Form, *http://www.flexiform.eu/),* with data extraction and review completed and confirmed in 2010 by at least two members of each center.

### Representative centers

The countries participating in the study were classified according to the World Bank classification as high-income countries (HIC) – Czech Republic and Slovakia; upper-middle income countries (UMC) – Hungary and Romania; lower-middle income countries (LMC) – Ukraine; and low-income countries (LIC) – none (*http://data.worldbank.org/news/2015-country-classifications*). Population based statistical data for each country were obtained from Eurostat, the statistical office of the European Union (*http://ec.europa.eu/eurostat*) or from the national statistical offices: Czech (*www.czso.cz/csu/czso/home*), Hungarian (*www.ksh.hu/docs/hun/eurostat_tablak/index.html*), Romanian (*www.indexmundi.com/romania/*), Slovakian (*www.slovak.statistics.sk*/), and Ukrainian (*www.ukrstat.gov.ua/*). Therefore, for the regression model, site (hospital) based data and the national or sub-national population based data were used.

### Statistical analysis

The data were analyzed using SAS software (SAS Institute, Cary, NC, USA) for both univariate and multivariate analysis to investigate and compare the relationship of most recognized risk factors of pregnancy with preterm birth. The ten most momentous variables based on relevant preterm birth risk literature and data availability were selected: history of smoking, pre-pregnancy diabetes, chronic hypertension, current diabetes, preeclampsia, progesterone use, current smoking, body mass index (BMI), iron use, and anemia during pregnancy. Univariate analysis was used to examine the relationship between preterm birth (binary variable) and each predictor separately. Multivariate stepwise logistic regression was used to examine the prediction of preterm birth by testing selection from all of the predictors into one model. Stepwise logistic regression was used to screen a large number of variables and to fit multiple logistic regression equations simultaneously.

## Results

Out of total 37 661 births, 33 794 were term and 3867 were preterm births. Mean percentage of preterm birth was 10.27, maternal age for preterm births was 29.30 ± 5.93 years, and mean maternal age for term births was 28.54 ± 5.39 years (Table 1). The preterm birth rate varied from 4.86% in Slovakia to 16.5% in Hungary (Pecs). Out of 94 total parameters in three questionnaires, only 34 were recorded in at least three centers and 19 in all the data sets. These data gaps represent lack of uniformity in data collection and patient data recording systems at the participating hospitals. Thus only ten factors suggesting variation between term and preterm birth from all centers could be further analyzed using both the univariate and multivariate model for preterm birth.

**Table 1:** Mean maternal age and total number of term and preterm births in each center for three years (except Hungary (Pecs) for 2 years)*

Center	% Preterm Births	Total (N)	Preterm (N)	Term (N)	Maternal age Mean±SD	
					preterm	term
**Czech**	10.67	5483	585	4898	30.40±5.45	30.28±4.81
**Hungary (Budapest)**	12.75	5857	747	NR	31.10±5.17	NR
**Hungary (Pecs)**	16.53	4137	684	3453	30.37±5.70	30.18±5.42
**Romania**	13.26	8076	1071	7005	28.06±6.31	27.03±5.59
**Slovakia**	4.86	7256	353	6903	29.65±5.64	29.67±4.94
**Ukraine**	6.23	6852	427	6425	26.23±5.50	26.79±5.06
**Total/average**	10.27	37661	3867	33794	29.30±5.93	28.54±5.39

*NR – data not recorded; SD – standard deviation.

The relationship between preterm birth and each predictor was analyzed initially by univariate analysis of the most significant risk factors (Table 2). For Romania, none of the variables were statistically significant to be included in univariate or multivariate model as predictors of preterm birth.

**Table 2:** Univariate analysis of the data for most significant risk factors for preterm birth at all sites. Blanks (-) indicate the missing data*

S.NO		Czech republic			Hungary (Budapest)			Hungary (Pecs)			Romania			Slovakia			Ukraine		
	Variable	% PTB	% TB	*P*	% PTB	% TB	*P*	% PTB	% TB	*P*	% PTB	% TB	*P*	% PTB	% TB	P	% PTB	% TB	*P*
**1**	**Body mass index (mean**±SD**)**	23.1 ± 4.7	27.1 ± 5.0	0.001	-	-	-	24.1 ± 5.3	23.8 ± 5.0	0.260	23.4 ± 3.8	23.4 ± 3.8	0.980	22.6 ± 4.5	22.8 ± 4.0	0.390	26.2 ± 2.8	26.4 ± 3.4	0.007
**2**	**History of smoking**	21.2	10.9	0.001	1.47	0.4	0.001	-	-		45.0	43.0	0.230	10.8	8.5	0.140	2.6	0.4	0.0001
**3**	**Current smoking**	17.1	7.8	0.001	1.47	0.23	0.001	15.85	7.03	0.000	-	-	-	10.2	7.8	0.100	2.1	0.2	0.001
**4**	**History of diabetes**	1.4	1.0	0.500	0.94	0.27	0.010	1.8	0.4	0.000	-	-	-	0	0.4	0.210	0	0.2	0.350
**5**	**Current diabetes**	8.9	7.8	0.370	8.57	5.97	0.008	9.7	7.7	0.090	3.8	3.7	0.860	11.9	8.6	0.040	0.0	0.2	0.350
**6**	**History of hypertension**	1.9	1.3	0.280	2.5	1.2	0.002	2.2	1.36	0.100	2.2	2.4	0.710	4.2	0.6	0.001	4.0	0.7	0.001
**7**	**Preeclampsia**	1.7	1.2	0.330	16.33	4.47	0.001	16.1	5.8	0.001	4.9	4.9	0.990	11.1	2.4	0.001	14.5	4.8	0.001
**8**	**Progesterone use**	9.1	5.2	0.001	1.2	0.4	0.001	-	-	-	17.7	17.7	0.970	5.7	6.9	0.350	43.8	3.6	0.001
**9**	**Anemia**	7.4	11.1	0.006	44.4	11.5	0.001	5.0	7.0	0.060	18.5	18.8	0.820	56.9	35.1	0.001	16.9	10.5	0.001
**10**	**Iron use**	7.9	11.1	0.020	44.4	10.5	0.001	17.3	14.5	0.050	36.5	36.2	0.850	57.5	40.7	0.001	16.9	10.5	0.001

*SD – standard deviation; PTB – preterm birth; TB – term birth.

BMI was a significant predictor of preterm birth in Czech Republic (*P* < 0.0001), Slovakia (*P* < 0.040), and Ukraine (*P* < 0.001), while data were missing for Hungary (Budapest). History of smoking or current smoking was significant in four centers (*P* < 0.0001 each respectively) (except Romania and Slovakia) and was higher in the preterm birth group. At one site only, Czech Republic, the history of diabetes and current diabetes were significant predictors (*P* < 0.020). Only Czech Republic, Slovakia, and Ukraine recorded higher rates of chronic hypertension in the preterm birth group. Preeclampsia was significantly higher in the preterm birth group for 4 centers (*P* < 0.001 each respectively) (except Czech Republic and Romania). Iron use was also significantly higher in Hungary (Budapest) (*P* < 0.0001), Slovakia, (*P* < 0.020), and Ukraine (*P* > 0.030). In Czech Republic, Hungary (Budapest), and Ukraine, progesterone use was significantly elevated in preterm birth group (*P* < 0.0001, *P* < 0.001, *P* < 0.0001 respectively). Anemia was a significant predictor in Czech Republic (*P* < 0.003) and Ukraine (*P* < 0.001) (Table 3).

**Table 3:** Multivariate logistic regression analysis with adjusted risk ratios of the preterm birth model. Blanks (-) indicate the missing data or non-significant *P* value

S. NO		Czech republic				Hungary (Budapest)				Hungary (Pecs)				Romania				Slovakia				Ukraine			
	Variable	% PTB	% TB	Adj RR (95% CI)	*P*	% PTB	% TB	Adj RR (95% CI)	*P*	% PTB	% TB	Adj RR (95% CI)	*P*	% PTB	% TB	Adj RR (95% CI)	*P*	% PTB	% TB	Adj RR (95% CI)	*P*	% PTB	% TB	Adj RR (95% CI)	*P*
**1**	**Body mass index (mean**±SD**)**	**23.1 ±4.7**	**27.1 ± 5.0**	**0.8 (0.8, 0.8)**	**0.0001**	**-**	**-**	**-**	**-**	**24.1 ± 5.3**	**23.8 ± 5.0**	**-**	**-**	**23.4 ± 3.8**	**23.4 ± 3.8**	**-**	**-**	**22.7 ± 4.5**	**22.5 ± 4.0**	**1.0 (0.9, 1)**	**0.04**	**26.2 ± 2.8**	**26.4 ± 3.4**	**0.9 (0.91,0.98)**	**0.001**
**2**	**History of smoking**	**21.2**	**10.9**	**2.4** **(1.9, 3.0)**	**0.0001**	**1.47**	**0.4**	**-**	**-**	**-**	**-**	**-**	**-**	**45**	**43**	**-**	**-**	**10.8**	**8.5**	**-**	**-**	**2.6**	**0.4**	**-**	**-**
**3**	**Current smoking**	**17.1**	**7.8**	**-**	**-**	**1.47**	**0.23**	**6.6 (2.7, 16.2)**	**0.0001**	**15.85**	**7.03**	**2.6 (1.98,3.32)**	**0.0001**	**-**	**-**	**-**	**-**	**10.2**	**7.8**	**-**	**-**	**2.1**	**0.2**	**11.6 (3.8, 35.6)**	**0.001**
**4**	**Current diabetes**	**1.4**	**1**	**-**	**-**	**0.94**	**0.27**	**-**	**-**	**1.8**	**0.4**	**-**	**-**	**-**	**-**	**-**	**-**	**0**	**0.4**	**-**	**-**	**0**	**0.2**	**-**	**-**
**5**	**History of diabetes**	**8.9**	**7.8**	**1.7 (1.2, 2.3)**	**0.002**	**8.57**	**5.97**	**-**	**-**	**9.7**	**7.7**	**-**	**-**	**3.8**	**3.7**	**-**	**-**	**12.1**	**7.7**	**-**	**-**	**0**	**0.2**	**-**	**-**
**6**	**History of hypertension**	**1.9**	**1.3**	**2.9 (1.4, 6.0)**	**-**	**2.5**	**1.2**	**-**	**-**	**2.2**	**1.36**	**-**	**-**	**2.2**	**2.4**	**-**	**-**	**4.8**	**1**	**4.5 (2.3, 8.9)**	**0.001**	**4**	**0.7**	**4.2 (1.9, 9.0)**	**0.003**
**7**	**Preeclampsia**	**1.7**	**1.2**	**-**	**-**	**16.33**	**4.47**	**3.4 (2.6, 4.4)**	**0.0001**	**16.1**	**5.8**	**3.2 (2.43,4.10)**	**0.0001**	**4.9**	**4.9**	**-**	**-**	**11.1**	**2.8**	**4.3 (2.9, 6.5)**	**0.0001**	**14.5**	**4.8**	**3.9 (2.8, 5.5)**	**0.001**
**8**	**Progesterone use**	**9.1**	**5.2**	**2.1 (1.5, 2.9)**	**0.0001**	**1.2**	**0.4**	**3.0 (1.3, 7.0)**	**0.01**	**-**	**-**	**-**	**-**	**17.7**	**17.7**	**-**	**-**	**0**	**5.5**	**-**	**-**	**43.8**	**3.6**	**21.4 (16.8, 27.3)**	**0.0001**
**9**	**Anemia**	**7.4**	**11.1**	**0.6 (0.4, 0.8)**	**0.003**	**44.4**	**11.5**	**-**	**-**	**5**	**7**	**-**	**-**	**18.5**	**18.8**	**-**	**-**	**60.3**	**32.8**	**5.1 (2.7, 9.8)**	**0.001**	**16.9**	**10.5**	**-**	**-**
**10**	**Iron use**	**7.9**	**11.1**	**-**	**-**	**44.4**	**10.5**	**6.3 (5.3, 7.5)**	**0.0001**	**17.3**	**14.5**	**-**	**-**	**36.5**	**36.2**	**-**	**-**	**60.3**	**38.6**	**0.4 (0.2, 0.9)**	**0.02**	**16.9**	**10.5**	**1.4 (1.0, 1.9)**	**0.03**

*SD – standard deviation; PTB – preterm birth; TB – term birth; CI – confidence interval; Adj RR – adjusted risk ratio.

From the multivariate model, 9 of the 10 factors chosen for study were predictive of preterm birth across all these European centers. Only history of diabetes was not predictive presumably because of its low prevalence in all sites. The pattern seen for the various countries was similar. Preterm birth was at least 2.4 times more likely with smoking (history or current) after adjusting for other covariates for 4 out of 5 sites that had data. It was at least three times more likely with preeclampsia after adjusting for other key factors at these sites. Similarly, it was at least 2.9 times more likely with hypertension after adjusting for covariates in 3 out of 5 sites. Preterm birth showed inverse relationship with the significant predictor BMI with adjusted risk ratio of 0.8 to 1.0 in 3 sites. Iron use and anemia, though significant predictors of preterm birth, indicated mixed patterns for relative risk ratio (Table 3).

### Cumulative data analysis in relation to the national rates

**Figure 1:**
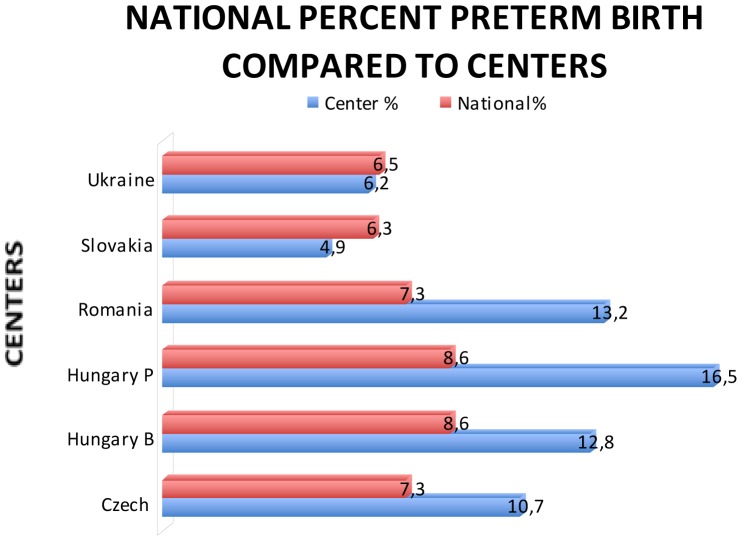
National percent of preterm birth vs six centers: Czech Republic, Hungary (Pecs and Budapest), Romania, Slovakia, and Ukraine.

**Figure 2:**
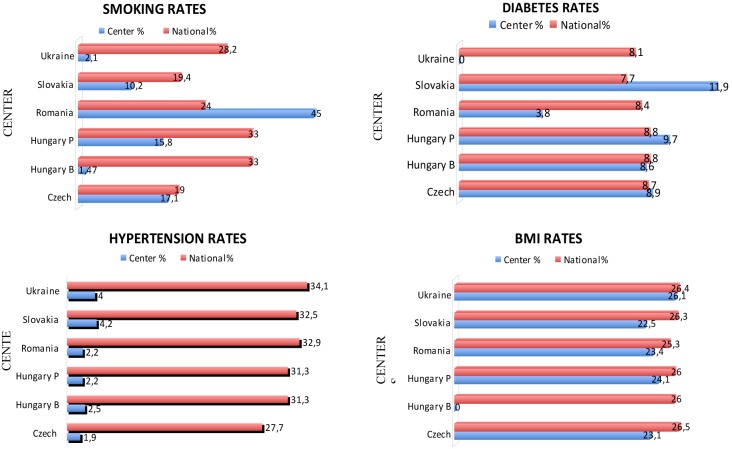
Significant historical risk factors of preterm birth (history of smoking, history of diabetes, hypertension, and body mass index) in relation to National Statistics. Very low values or missing values from the centers reflect the missing data or incomplete data collection.

Data outcome of SAS analysis and PTB frequencies for each country were calculated in relation to the national average (Figure 1). Preterm rates were higher than the national data in 4 University Hospitals. Ukraine and Slovakia did not show such a difference. Romania and Hungary (Pecs) had almost two times higher preterm birth rate than the national one since the former is the high-risk pregnancy center and the latter is a University Hospital and regional tertiary center for preterm birth. Four of the most commonly associated risk factors: BMI, history of smoking, history of diabetes, and hypertension were also compared with national demographics of each country (Figure 2). Smoking rates reported in all the centers except Romania were lower than the national levels. Hypertension rates were extremely low in all the centers. BMI was not recorded in Hungary (Budapest), while the rest of the centers reported comparable rates to the national stats. Diabetes rates were high in Slovakia and Hungary (Pecs) centers but not recorded in Ukraine.

## Discussion

Estimating preterm birth rates and associating risk factors (eg, smoking, prior preterm birth, hypertension, diabetes etc.) are essential for determining global incidences of PTB and for developing clinical interventions to reduce the risk of premature birth and related long-term cardiovascular, neurological, and metabolic disorders of both mother and child. Significant risk factors for preterm birth in this study were smoking, diabetes, and chronic hypertension. These factors in conjunction with prior preterm birth and preeclampsia have been strongly correlated with preterm birth and high risk of morbidity due to different mechanisms (1,6). Prior preterm birth in our study was recorded only in Czech Republic (2.9%), Slovakia (0.7%), Ukraine (2.7%), and Hungary (Pecs) (3.5%). The missing data from Romania and Hungary (Budapest) made it difficult to analyze and interpret its significance conclusively in the total population enrolled in this study.

The study identified BMI as one of the most significant factors associated with preterm births in CEE. It is important to consider contextual differences in obesity and preterm birth in the region. The interaction between pre-pregnancy BMI and weight gain during pregnancy could not be assessed in this analysis. Our computation did not take into account the duration of pregnancy. Calculating the weekly weight gain rate to adjust for the length of pregnancy was not possible due to insufficient data on the timing of the first prenatal care. BMI is usually an indicator of nutrition status during pregnancy and has been associated with preterm birth in US and some other developed countries (1). Obese women are more likely to develop diabetes and preeclampsia, causing maternal complication enhancing the odds of preterm delivery (16,17).

Smoking rates of each center were compared to the national rates, except in Ukraine. Four out of 5 sites indicated lower rates of smoking than the national data. This finding may suggest that insufficient attention in the network is being directed toward collecting information on this very important potential behavioral issue. A point to remember is that women tend to hide smoking during pregnancy. Alternatively, women in the network may have been advised not to smoke during pregnancy. There are also the latent factors to help or hinder smoking cessation, enhancing the tendency to conceal it during pregnancy. Smoking was also a strong predictor of preterm birth in our study, perhaps by instigating systemic inflammation and vasoconstriction by nicotine, carbon monoxide, and other toxins in cigarette smoke (18).

A retrospective study by Rebarber et al reported an adverse effect of progesterone use (19). This study showed that the incidence of gestational diabetes was 12.9% in women treated with progesterone (n = 557) compared with 4.9% in controls. Since ovarian progesterone deficiency appears to be associated with fetal-placental (including membranes and decidua) dysregulation, progesterone has been used to prevent preterm labor and recurrent preterm delivery (20). Although progesterone is widely used in the early phase of pregnancy as a precaution against threatened abortion, our multivariate analysis indicated that its use was statistically significant in Czech Republic, Hungary (Budapest), and Ukraine sites only.

Iron supplementation during pregnancy was a significant risk factor for preterm birth in Hungary (Budapest), Slovakia, and Ukraine. In addition, the use of iron supplements was higher in women with preterm birth than with term birth at all sites except the Czech Republic. It is worth mentioning that there are no clear recommendations across the region regarding iron supplementation during pregnancy. Variation in dosage is anywhere from 60-240 mg/d prescribed from beginning of pregnancy or at the time of first clinic visit, which might be any time during pregnancy. Also, there was no uniformity in using Ferrous or Ferric iron in the centers. As iron readily shuttles between the reduced ferrous (Fe^2+^) and the oxidized ferric (Fe^3+^) forms, only catalytic amounts can cause disruption of the cellular macromolecules and redox equilibrium. Importantly, it has been reported (21) that iron during pregnancy could be toxic to the developing brain in mice.

The participating sites reported very low rates of hypertension compared to the national data perhaps due to the insufficient data collection. The importance of assessing the incidence of hypertension in women during the childbearing years should not be overlooked. However, in this young population, the incidence may be low, although comparing the incidence with the national data without controlling for age and other factors may be an issue. The average incidence of diabetes was 8.6% among 5 sites reporting, with the lowest rate in Romania (3.8%), the highest rate in Slovakia (12.1%), and insufficient data from Ukraine, suggesting that different risk factors influence the rates of preterm birth in different countries in the region (22,23).

Preterm birth has been recognized as a worldwide problem, particularly in LMC where its rate is around 25% compared to 5% in HIC. Moreover, in developing countries, 15% of all neonatal deaths are caused by prematurity and its complications (24,25). It has been emphasized that perinatal distress had an effect on cardiovascular and pathophysiological changes in pregnant women and fetuses/newborns in LMC (26)

The relative neglect of preterm birth is linked to data gaps in UMC and LMC and even HIC in Central and Eastern Europe, and consequential prematurity, neonatal morbidity, and mortality. The new estimates shown in this report make a substantial contribution to addressing this deficiency. Widely held perceptions that effective care of the preterm baby requires costly interventions well beyond the health budgets of most LMCs, coupled with concerns that greater attention to preterm birth will draw needed funding away from other devastating maternal and perinatal health problems, have also contributed to the reluctance of policy makers to focus on the problem of preterm birth as global priority (14,23,24,27).

There are many reasons for the inadequacies of preterm birth-related epidemiology in CEE, including patient management by primary health care services, incomplete health-related statistics, and health information systems, lack of preterm birth surveillance registries or imperfect coordination among existing registries and reliance on hospital based rather than population-based studies (23,28,29). Estimates of the preterm birth rate are influenced by factors including the procedures to determine gestational age and national differences in birth registration processes. More effort is needed to influence the content of midwifery and medical pre- and in-service education and to establish gestational age assessment as an integral component of routine care (13,27) and nationwide implementation of electronic medical records. Although some comprehensive data collection questionnaires have been developed by March of Dimes (*http://www.marchofdimes.org*/), International PREterm BIrth Collaborative (PREBIC *www.prebic.net*) of the World Health Organization, and Department of Reproductive Health and Research (*http://www.who.int/reproductive-health/index.htm*), efforts to harmonize the data collection are equally vital to the national, regional, and global programs. Excellent recommendations to improve data have been suggested by Lawn et al (24)

This study is the first attempt to investigate term and preterm birth using a large collaborative approach in the region and it is part of the learning process with shortcomings of retrospective data collection. One of the limitations was the type of participating sites. Since RECOOP HST Association members are Universities and Academic Research Centers, the study could not provide comprehensive data of the participating CEE countries. We created and used standardized electronic data collection forms to gather information at all sites from paper medical records. Other limitations of the study include the method and limitation of data collection at different sites, and the sampling of one or two sites per country, which does not represent the entire country. We tried to validate the data with available national statistics but the major problem was the imperfect data in the paper based medical records.

During the retrospective data collection, it was impossible to gather information on the pre- pregnancy BMI from all sites, but still the study identified BMI as the most significant risk factor in the region. The correlation between obesity and diabetes is well known and the incidence of diabetes in the study is high and comparable to the national rates. However, smoking and high blood pressure are major public health issues in CEE although in the studied cohort the smoking rates and hypertension were lower than the national data. During the retrospective data collection, investigators could not have validated the documented smoking records and limited data were available on blood pressure measurements at the admission and during the hospitalization of the subjects.

In conclusion, smoking, BMI, preeclampsia, and hypertension were the most significant risk factors in the region. Comprehensive public health education aimed at behavioral modification and lifestyle change could affect smoking and BMI of the population, which is not only beneficial for the society but it is also likely to have a major effect on the overall rate of preterm birth. Thorough epidemiological data are crucial for any intervention to improve the birth outcomes in the region, so nationwide implementation of electronic birth registration among hospitals is imperative for an effective PTB intervention program.
